# Triazole-modified chitosan: a biomacromolecule as a new environmentally benign corrosion inhibitor for carbon steel in a hydrochloric acid solution†

**DOI:** 10.1039/c9ra00986h

**Published:** 2019-05-14

**Authors:** Dheeraj Singh Chauhan, M. A. Quraishi, A. A. Sorour, Sourav Kr. Saha, Priyabrata Banerjee

**Affiliations:** Center of Research Excellence in Corrosion, Research Institute, King Fahd University of Petroleum and Minerals Dhahran 31261 Saudi Arabia mumtaz.quraishi@kfupm.edu.sa dheeraj.chauhan@kfupm.edu.sa; Surface Engineering & Tribology Group, CSIR-Central Mechanical Engineering Research Institute Mahatma Gandhi Avenue Durgapur 713209 West Bengal India

## Abstract

In this work, a new inhibitor, triazole modified chitosan, was synthesized for the first time following chemical modification of chitosan using 4-amino-5-methyl-1,2,4-triazole-3-thiol. The newly synthesized biopolymer (CS–AMT) was characterized using FTIR and NMR, and then it was evaluated as an inhibitor against corrosion of carbon steel in 1 M hydrochloric acid. The corrosion testing and evaluation were performed thoroughly employing the weight loss method, electrochemical measurements and surface analysis. A maximum corrosion inhibition efficiency of >95% was obtained at 200 mg L^−1^ concentration of inhibitor. The adsorption of inhibitor obeyed the Langmuir isotherm and showed physical and chemical adsorption. The electrochemical study *via* impedance analysis supported the adsorption of the inhibitor on the surface of carbon steel, and the potentiodynamic polarization indicated a mixed type of inhibitor behavior with cathodic predominance. To get a better insight on the interaction of inhibitor molecules with the metal surface, a detailed theoretical study was performed using DFT calculations, Fukui indices analysis and molecular dynamics (MD) simulation. The DFT study showed a lower energy gap of CS–AMT and the MD simulations showed an increased binding energy of CS–AMT compared to the parent chitosan and triazole moieties thereby supporting the experimental findings.

## Abstract 2

A novel derivative of chitosan is evaluated as an environment-friendly corrosion inhibitor for carbon steel in hydrochloric acid solution.

## Graphical abstract

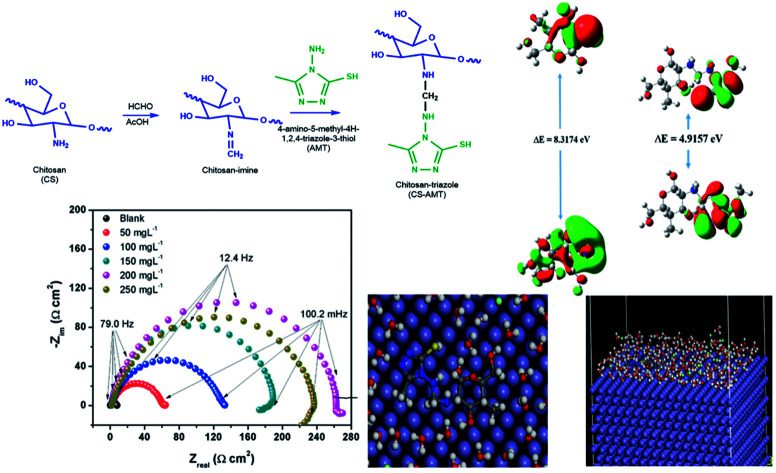

## 1. Introduction

Carbon steel is widely used in the petroleum industries due to its cost-effectiveness and high mechanical strength. To remove unwanted scales and salt deposits and improve oil recovery, processes such as oil well acidization, acid pickling, and acid descaling are carried out, using mineral acids *e.g.* hydrochloric acid.^1–4^ These acids cause a corrosive attack and damage to the carbon steel surface. This damage engenders high cost for renovation and replacement of various equipment and causes public and environmental loses.^5,6^ One of the most common strategies to counter this problem is the introduction of organic compounds as corrosion inhibitors to the acid solution.^1,7,8^ The adsorption of organic inhibitor molecules on the metal surface prevents direct contact between the metal surface and acid solution. However, most of the organic-based corrosion inhibitors are expensive and toxic to human beings and environment. For this reason, researchers are currently focusing on the development of cost-effective and “green” corrosion inhibitors with high efficiency.^1,7,8^

In this context, the polymeric molecules having a high molecular weight and thus a greater surface coverage have proved to be promising corrosion inhibitors. Accordingly, carboxymethyl cellulose, Tapioca Starch, Gum Arabic and Chitosan have been reported as promising corrosion inhibitors.^9–11^ Chitosan is a polysaccharide bearing β-(1-4)-linked *N*-acetyl-d-glucosamine units in the chain. It is a hydrophilic biomacromolecule extracted by *N*-deacetylation of chitin which constitutes the exoskeletons of crustaceans, fungi and simple arthropods following alkaline treatment.^12^ Chitosan is widely reported for its antibacterial/antifungal properties and is used as a drug-carrier in modern therapeutics.^12–14^ Chitosan is also reported for a number of applications in the paper, textile and food industry.^12,15^ The corrosion inhibitive behavior of chitosan is attributable to the –NH_2_ and –OH groups which could facilitate its adsorption over the metal surface. Therefore, a number of researchers have investigated pure and chemically functionalized chitosan as inhibitors against corrosion of a variety of metallic materials.^16–18^ However, the application of chitosan and its derivatives suffers from poor solubility in the testing medium which affects the anti-corrosion behavior.

Earlier we have reported the corrosion inhibition behavior of chitosan on carbon steel in 1 M sulphamic acid and observed the synergism with potassium iodide (KI).^17^ The interesting findings prompted us to perform the chemical functionalization of chitosan to improve the corrosion inhibition performance. Accordingly, thiosemicarbazide, thiocarbohydrazide and polyethylene glycol^1,8,19^ were used to prepare chemically modified chitosan which was studied as the corrosion inhibitor for carbon steel in acid solutions. Further, we firstly reported the microwave-assisted synthesis of chitosan Schiff bases and used them as inhibitors against carbon steel corrosion in 1 M HCl.^7^ In continuation of our research work on the development of green corrosion inhibitors,^20–23^ we herein report the synthesis and characterization of chitosan functionalized with 4-amino-5-methyl-1,2,4-triazole-3-thiol (AMT). The selection of triazolic compound is based on the fact that the triazoles are well known for their corrosion inhibition behavior.^24–27^ The excellent corrosion protection ability of triazoles arises due to the presence of heterocyclic ring containing three nitrogen atoms along with the presence of π bonds. The present triazole, *i.e.*, AMT, contains an –SH group which is likely to improve the corrosion inhibition behavior. Earlier we have used AMT as an inhibitor against the corrosion of copper in 1 M HCl.^24^

The newly developed corrosion inhibitor is a combination of two inhibitors, *i.e.*, chitosan and AMT. While triazoles have been most commonly used for copper, chitosan-based inhibitors have been used for carbon steel. Either of them alone is not effective for carbon steel in hydrochloric acid solution. Accordingly, in the present work, it was considered worthwhile to test the newly synthesized chitosan–AMT (CS–AMT) to study the corrosion inhibition performance of carbon steel in 1 M HCl solution by weight loss and electrochemical measurements as well as surface analysis. In addition, quantum chemical calculations using density functional theory (DFT) and Fukui analysis were performed to estimate the reactivity parameters of the inhibitor theoretically.

## 2. Experimental

### 2.1 Materials used

Chitosan (Mol. wt 190 000), and formaldehyde (36%) were purchased from Aldrich, USA. All the other chemicals were purchased from Aldrich and had analytical grade purity. The carbon steel samples used in the present study had a chemical composition in wt%: C 0.076%, Cu 0.135%, P 0.012%, Si 0.026%, Mn 0.192%, Cr 0.050%, Ni 0.050%, P 0.012%, Al 0.023%, balance Fe. The samples were cut into size 2.5 cm × 2.0 cm × 0.025 cm for the weight loss study. For the electrochemical measurements, the samples used had dimensions of 8 cm × 1.0 cm × 0.025 cm having 1 cm^2^ unmasked surface. After abrading with emery paper having grades 400 to 1200, the metal samples were washed with double distilled water. Then the samples were degreased using acetone, air-dried, and kept in desiccators. The solutions of 1 M hydrochloric acid were made by dilution of analytical grade HCl (Aldrich) with double distilled water.

### 2.2 Synthesis of CS–AMT

**Fig. 1:**
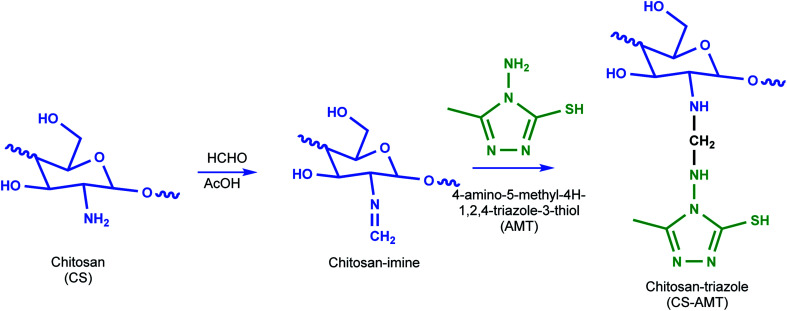
Schematic representation of the synthesis of CS–AMT.

**Fig. 2:**
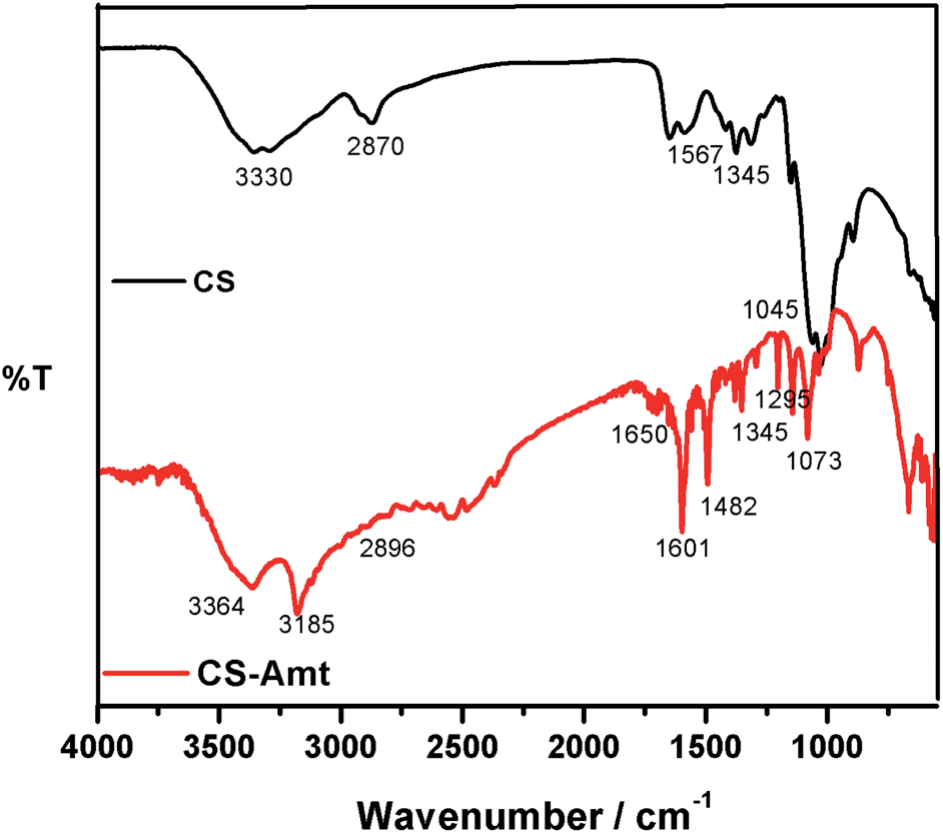
FTIR spectra of CS and the functionalized derivative CS–AMT.

The synthesis of 4-amino-5-methyl-1,2,4-triazole-3-thiol (AMT) was performed as described earlier.^28^ The synthesis of chitosan–triazole was performed as a modified procedure from the earlier reported synthesis of chitosan–thiosemicarbazide and chitosan–thiocarbohydrazide.^1^ Briefly, chitosan (1.0 g) was introduced to a dilute solution of acetic acid (30 mL) which resulted in a viscous solution. AMT (0.975 g) was dissolved in a mixture of ethanol and water (60 : 40 ratio) resulting in a 20 mL solution and added to the solution of chitosan followed by stirring to result in a clear solution. Formaldehyde (1 mL) was added after 45 min, and the solution was subjected to reflux at 80 °C for 12 h to obtain a white gel. The product (CS–AMT) was treated with aqueous NaOH for neutralization to afford the precipitate. Further filtration and washing with distilled water resulted in the formation of a pale yellow (CS–AMT) product. The synthesis procedure is schematically represented in Fig. 1. The samples were characterized using a Nicolet iS5 FTIR having an iD5 ATR in the range 4000–400 cm^−1^. The FTIR spectra are shown in Fig. 2. The samples were dissolved in D_2_O, and the ^1^H NMR analysis was carried out using a Bruker 500 MHz NMR spectrometer shown in Fig. S1.† IR (*ν*_max_/cm^−1^): 3364 (N–H), 2896 (C–H), 1650 (C=N, imine), 1601 (C=N, triazole), 1345 (C–N), 1295 (C=S), 1073 (C–O). ^1^H NMR (500 MHz, D_2_O): *δ*_H_ (ppm), 1.7 (HAc), 2.0 (NH), 7.50 (NCH), 2.37 (CH_3_), 2.6 (triazole CH); m.p. 240 °C.

### 2.3 Corrosion tests

#### 2.3.1 Gravimetric study

The gravimetric experiments were undertaken in accordance with the ASTM standards.^30^ The cleaning of the metal samples after immersion tests was carried out using Clarke solution following standard method.^29–32^ The CS–AMT exhibited a solubility of 2500 mg L^−1^ in 1 M HCl. Stock solutions of 1000 mg L^−1^ were prepared and diluted with 1 M HCl to prepare the different concentrations of CS–AMT. The experiments were twice repeated, and the obtained weight loss data were used to compute the corrosion rate (*C*_R_; mg cm^−2^ h^−1^) as follows:^1,7,8^1
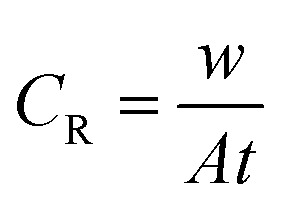
where *w* denotes the weight loss (mg), *A* represents the sample area (cm^−2^) and *t* symbolizes the exposure time (h^−1^). The corrosion inhibition efficiency (*η*%) and the surface coverage (*θ*) were be calculated from the *C*_R_ values:^1,4,7,8^2
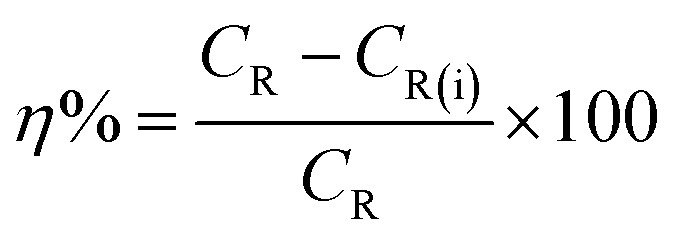
3
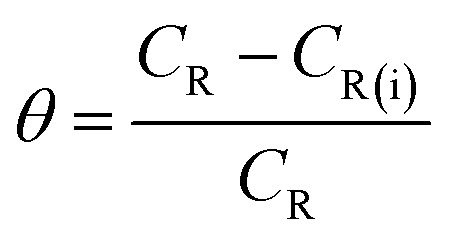
where *C*_R_ and *C*_R(i)_ denote the obtained corrosion rates in the absence and presence of CS–AMT.

#### 2.3.2 Electrochemical measurements

A Gamry Reference 600 was used to carry out the electrochemical analyses. The experimental data simulated and analyzed using the GamryEChem Analyst 5.0.^1,7,8^ A single compartment, three-electrode glass cell was used for the electrochemical studies. The assembly comprised of a carbon steel sample as a working electrode, a saturated calomel electrode as the reference (SCE) and a graphite rod as the counter electrode. Before measurements, the working electrode was dipped in the test electrolyte of 1 M HCl for 30 min in order to attain a steady open circuit potential (*E*_OCP_). The electrochemical impedance spectroscopy (EIS) was carried out at *E*_OCP_ from 100 kHz to 0.01 Hz frequency range using a 10 mV peak to peak AC signal. The potentiodynamic polarization (PDP) measurements were performed by sweeping the potential of the working electrode in the range ±250 mV *vs. E*_OCP_ at a scan rate of 0.1 mV s^−1^.

#### 2.3.3 Surface study

FTIR measurements were employed to analyze the adsorption of CS–AMT on carbon steel surface using the attenuated total reflectance (ATR) mode. A ZiessEvo 50 XVP scanning electron microscopy was employed to study the surface morphology of the carbon steel samples in the absence and the presence of the CS–AMT.

### 2.4 Computational studies

#### 2.4.1 DFT calculations

DFT calculations were carried out using the Gaussian 09 (ref. 33) as described earlier.^1^ The calculations were carried out at the B3LYP functional level using 6-31G (d, p) basis set.^34^ The calculations were performed in the aqueous phase using the integral equation formalism polarizable continuum model (IEFPCM) for neutral and protonated inhibitor molecules.^1,35^

#### 2.4.2 Fukui indices

The local reactivity was analyzed by the Fukui indices. The calculations were undertaken using the Dmol^3^ module in Material studio™ version 6.1 (Accelrys Inc.).^36^ The calculations were carried out at B3LYP level using double numerical with polarization (DNP) basis set.^36,37^ The Fukui function (*f*_k_) can be described as the first derivative of the electron density *ρ*(*r⃑*) in a constant external potential *ν*(*r⃑*) with respect to the number of electrons *N*:4
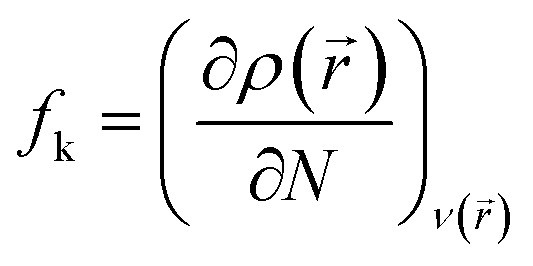


The Fukui functions were computed using the Finite Difference approximation method:^24^

5 *f*_k_^+^ = *q*_k_(*N* + 1) − *q*_k_(*N*) (nucleophilic attack)

6 *f*_k_^−^ = *q*_k_(*N*) − *q*_k_(*N* − 1) (electrophilic attack)

where *q*_k_ denotes the net charge on the atom. The *q*_k_(*N* + 1), *q*_k_(*N*) and *q*_k_(*N* − 1) symbolize the charge on the anionic, neutral and cationic moieties respectively.

#### 2.4.3 Molecular dynamics simulation

The molecular dynamics (MD) simulations were carried out using the discover module in Materials Studio™ 6.1 (ref. 36–38) to understand the orientation and adoption behavior of the studied inhibitors. Fe (1 1 0) surface was chosen considering its high stability and a packed surface, *i.e.*, its lower energy among the other Fe surfaces.^35^ MD simulations were performed in a simulation box of dimension (39.49 × 39.49 × 85.62 Å) with periodic boundary condition. Simulation box was constructed with three layers. The first layer is the Fe slab (constructed by the 10 layers of Fe), the second layer is the solution slab (containing the studied inhibitor molecule, 150 H_2_O molecules, 15 H_3_O^+^, 15 Cl^−^) and the third layer is the vacuum layer. Herein, the COMPASS force field was used in order to adjust the atomic coordinate of the studied molecules. The dynamic simulation was carried out by this force field.^36,37,39^ In this particular investigation, MD simulations were performed with NVT canonical ensemble for a simulation time of 100 ps with 1.0 fs time step at 298 K. The interaction energy (*E*_interaction_) between the iron surface and the inhibitor molecules is described as the difference between the total energy and the sum of energy of iron surface together with H_2_O, H_3_O^+^, Cl^−^ along with the single point energy of the inhibitor molecule.^36^ The negative of the interaction energy presents the binding energy of the inhibitor molecules as follows:^36,37,39^

7 *E*_binding_ = −*E*_interaction_

## 3. Results and discussion

### 3.1 Gravimetric studies

#### 3.1.1 Influence of concentration

**Fig. 3:**
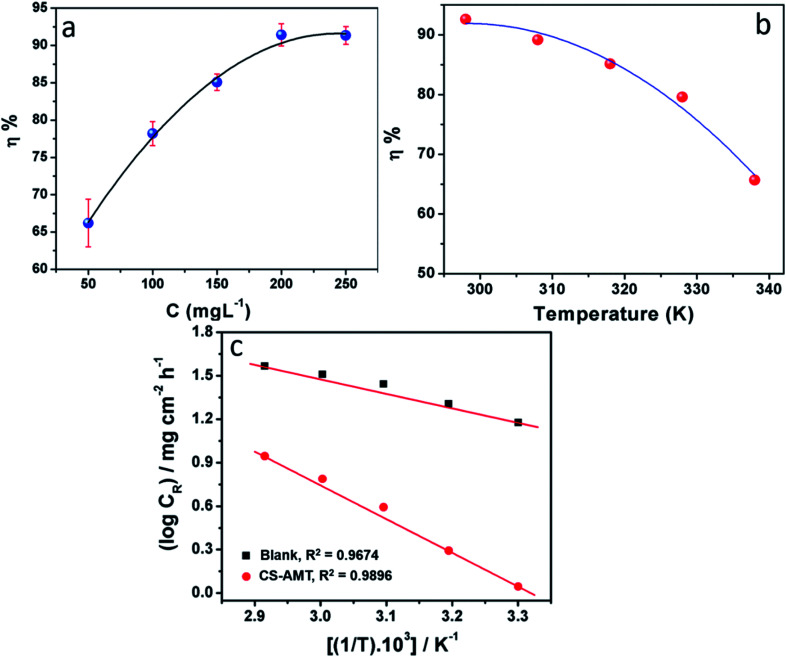
(a) Variation in corrosion inhibition efficiency (*η*%) with inhibitor concentration at 303 K. (b) Variation of inhibition efficiency (*η*%) with solution temperature (303–343 K) at an optimum concentration (200 mg L^−1^) of CS–AMT. (c) Arrhenius plots of the corrosion rate (*C*_R_) of carbon steel in 1 M HCl in the absence and presence of optimum concentration (200 mg L^−1^) of CS–AMT.

The effect of the different concentrations of CS–AMT on the inhibition performance was analyzed using the weight loss studies conducted in 1 M HCl by introducing the varying concentrations of CS–AMT from 50 mg L^−1^ to 250 mg L^−1^ to the test solution. Fig. 3a depicts the obtained results, and the calculated data are listed in Table 1. An obvious increase is observed in the inhibition efficiency with increasing concentrations of the inhibitor which indicates the adsorption of the inhibitor on the metal surface.^1,40,41^Table 1 lists the corresponding data which corroborates this finding and suggest an increment in the surface coverage and the percentage inhibition with an increase in the concentration of CS–AMT.

**Table 1:** Weight loss parameters obtained for MS in 1 M HCl in the absence and presence of different concentrations of CS–AMT

	Inhibitor conc. (mg L^−1^)	*C*_R_ (mg cm^−2^ h^−1^)	Surface coverage (*θ*)	*η*%
Blank	—	12.93 ± 0.02	—	—
CS–AMT	50	4.37 ± 0.04	0.6620	66.20
	100	2.82 ± 0.03	0.7819	78.19
	150	1.93 ± 0.06	0.8507	85.07
	200	1.11 ± 0.01	0.9142	91.42
	250	1.12 ± 0.02	0.9134	91.34

#### 3.1.2 Effect of temperature

The weight loss studies were performed in the temperature range of 303 to 343 K at 200 mg L^−1^ concentrations of CS–AMT for studying the effect of temperature on the inhibition efficiency. The results in Fig. 3b show that the *η*% exhibits a decreasing trend with the rise in temperature indicating that the inhibitor molecules undergo desorption from the metal surface. The elevation in temperature also causes a rise in the metal dissolution rate which causes an increase in the corrosion rate (*C*_R_) and a concomitant lowering in the corrosion protection. The Arrhenius equation can be applied to describe the relationship between the temperature and the *C*_R_:^1,7,8,42^8
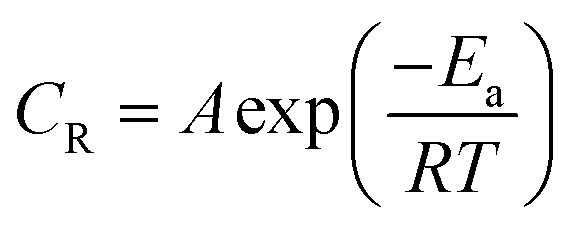
where, *A* symbolizes the Arrhenius pre-exponential factor, *E*_a_ denotes the energy of activation, *R* denotes the Universal gas constant and *T* denotes the temperature. A plot of log *C*_R_ and 1/*T* shows a straight line having a slope *E*_a_/2.303*R* (Fig. 3c). *E*_a_ for the blank and in the presence of CS–AMT were 20.23 and 44.92 kJ mol^−1^ respectively. It is obvious that the value of *E*_a_ in the presence of CS–AMT is considerably higher compared to that in its absence. A greater *E*_a_ value obtained in the presence of inhibitor is indicative of an increase in the thickness of the double layer, which leads to an increase in the activation energy barrier for the corrosion process.^43,44^ In literature, the observed increase in the activation energy values in the presence of a corrosion inhibitor is generally attributed as a result of the physical adsorption.^45^ However, the adsorption process cannot be solely classified as purely physical adsorption which is the first stage of adsorption nor it can be classified as purely chemical adsorption.^45^ In addition, the adsorption type cannot be decided purely on the basis of the trends in the *E*_a_ values due to the competitive adsorption with pre-adsorbed water molecules which also require some activation energy for their removal from the metal surface.^46^ Therefore, it can be inferred that, the adsorption of the CS–AMT on the steel surface in 1 M HCl solution takes place through physical as well as chemical mechanisms.^47^

#### 3.1.3 Adsorption isotherm

The calculated surface coverage values for the adsorption of inhibitor are plotted as a function of the varying concentration of inhibitor to derive an adsorption isotherm. This plot is commonly known as an adsorption isotherm is used to obtain information about the interaction between the molecules of a corrosion inhibitor and the metal surface in question.^2,4^ The experimentally obtained data were fitted to different adsorption isotherms among which the Langmuir isotherm provided the best fit and can be represented as:^1,7,8^

**Fig. 4:**
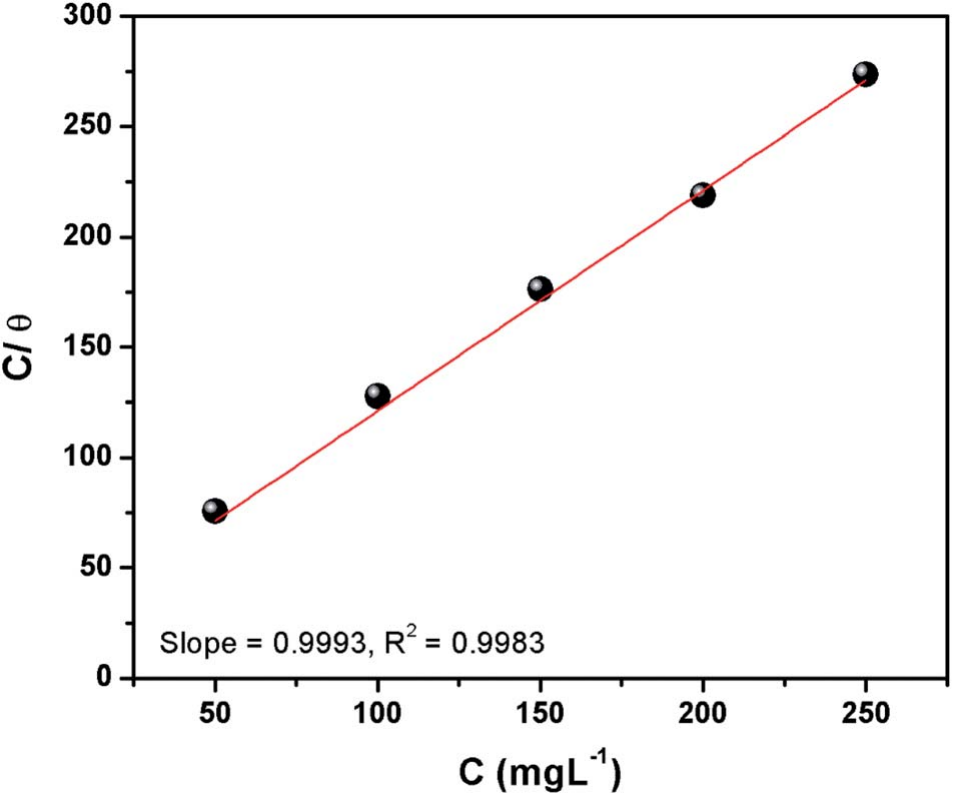
Langmuir isotherm plots for adsorption of CS–AMT on carbon steel surface in 1 M HCl.

9 *C*/*θ* = 1/*K*_ads_ + *C*

where *C* denotes the concentration of CS–AMT in mg L^−1^, *θ* symbolizes surface coverage and *K*_ads_ represents the equilibrium constant of adsorption. The *K*_ads_ value as obtained from the intercept of the isotherm was 65.3 L g^−1^. The Langmuir isotherm for adsorption of CS–AMT is shown in Fig. 4. Further, the standard free energy of adsorption Δ*G*^o^_ads_ can be computed from the following equation:^1,8^

10 Δ*G*^o^_ads_ = −*RT* ln(1000*K*_ads_)

The concentration of water in the solution is represented in 1000 g L^−1^. A value of Δ*G*^o^_ads_ below −20 kJ mol^−1^ indicates physical adsorption *via* electrostatic attraction and above −40 kJ mol^−1^ indicates chemisorption. In the present study, the calculated values of Δ*G*^o^_ads_ for CS–AMT is −28.4 kJ mol^−1^, respectively at the optimum inhibitor concentration (200 mg L^−1^). This suggests that the CS–AMT undergoes both physical and chemical adsorption.^1,2^

### 3.2 Electrochemical analysis

#### 3.2.1 Open circuit potential *versus* time

**Fig. 5:**
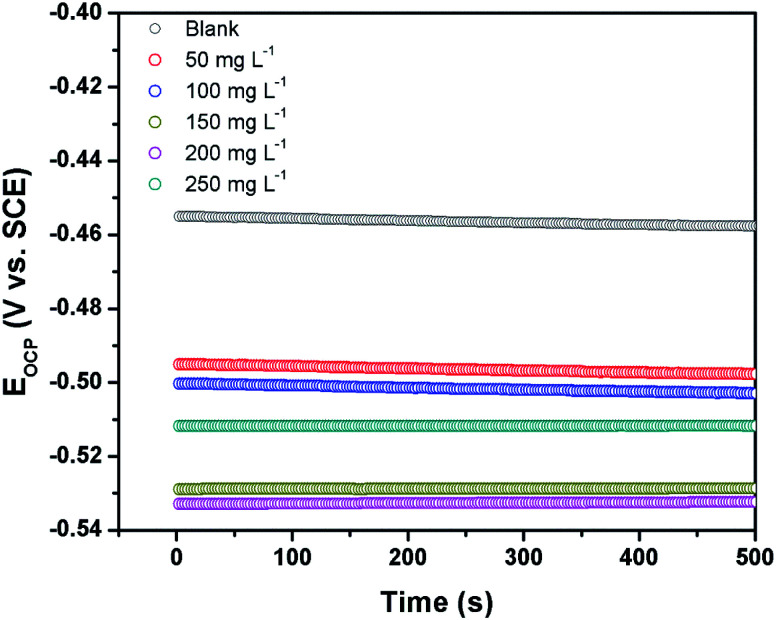
Open circuit potential *vs.* time curves for carbon steel surface in 1 M HCl the absence and the presence of different concentrations of CS–AMT.

The open circuit potential (OCP) refers to the potential of the working electrode with respect to that of the reference electrode in the absence of any externally applied potential or current. It is a prerequisite to run the OCP measurement prior to performing the EIS and the potentiodynamic polarization measurement. The OCP *vs.* time curves of the steel electrode immersed in 1 M HCl solution in the absence and the presence of different concentrations of CS–AMT are shown in Fig. 5. It can be observed that the steady-state OCP values, in the absence of the inhibitor are more negative compared to that of the blank steel sample. This shift of the OCP can be explained in terms of formation of a protective layer of inhibitor molecules on the metal surface.^4,48^

#### 3.2.2 Electrochemical impedance measurements

**Fig. 6:**
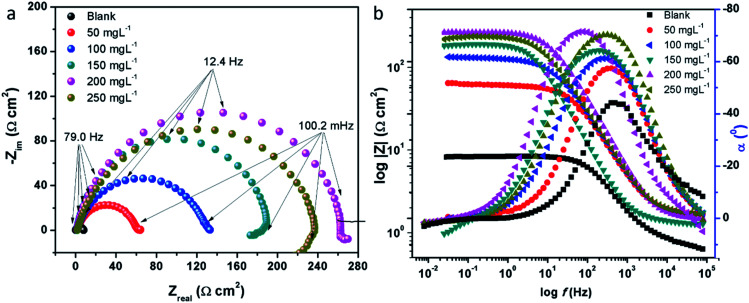
(a) Nyquist plots for carbon steel in 1 M HCl in the absence and presence of 50 to 250 mg L^−1^ concentration of CS–AMT at 308 K; (b) Bode (log *f vs.* log|*Z*|) and phase angle (log *f vs. α*^0^) plots for carbon steel in 1 M HCl.

The electrochemical impedance spectroscopy (EIS) can provide a rapid and convenient means to investigate the performance of inhibited metal surface.^6,49–52^ The EIS offers the major advantage in the ability to follow the corrosion behavior of metals with time. The impedance spectra of the carbon steel substrate in 1 M HCl solution without and with varying concentrations of CS–AMT are depicted in Fig. 6a in the form of Nyquist plots. The corresponding Bode and phase angle plots are shown in Fig. 6b. The Nyquist plots show depressed semicircles having one capacitive loop suggestive of the presence of single time constant.

**Fig. 7:**
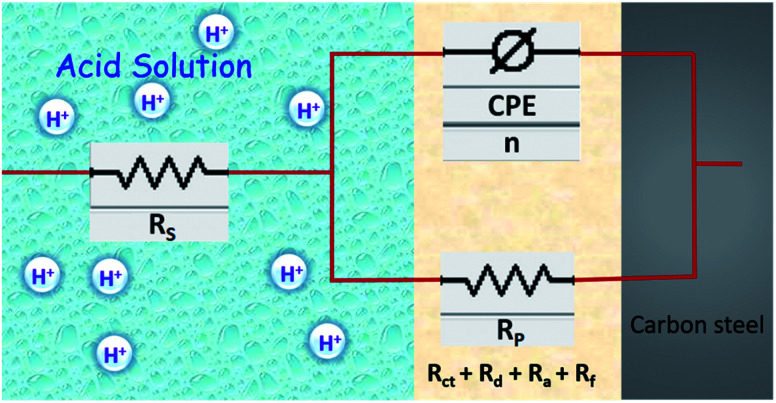
Schematic representation of double layer and equivalent circuit model used to fit the EIS data.

The electrochemical EIS parameters listed in Table 2 were calculated by fitting the obtained EIS data to the equivalent circuit shown in Fig. 7. The *R*_s_ symbolizes the collection of the resistance arising from the electrolyte and the electrical connections. The charge transfer resistance (*R*_ct_) conventionally describes the difference in the real impedance between the highest and the lowest frequencies.^6,50^ In cases where a metal electrode is immersed in an aggressive electrolyte and undergoes uniform corrosion, a number of additional resistance contributions must be taken into account such as the film resistance (*R*_f_), the resistance of the diffuse layer (*R*_d_), and the resistance from other accumulations (*R*_a_). The influence of the different accumulations can be observed as the “tails” or the elongations at the lower frequencies in the Nyquist diagrams.^1,22,53^ The collective contribution of the *R*_ct_ along with all these resistance contributions is known as the polarization resistance (*R*_p_).^6,50^

**Table 2:** Electrochemical impedance parameters in the absence and presence of different concentrations of CS–AMT

	Inhibitor conc. (mg L^−1^)	*R*_s_ (Ω)	*R*_p_ (Ω cm^2^)	*n*	*C*_dl_ (μF cm^−2^)	*η*%
Blank	0	0.66 ± 0.05	11.34 ± 0.08	0.830	486.36 ± 26.3	—
CS–AMT	50	0.79 ± 0.06	60.38 ± 1.11	0.876	59.62 ± 4.6	86.85
	100	0.87 ± 0.02	127.30 ± 1.09	0.886	53.26 ± 3.7	93.76
	150	0.83 ± 0.03	189.30 ± 2.30	0.854	43.96 ± 2.8	95.80
	200	0.78 ± 0.04	262.50 ± 3.02	0.894	39.25 ± 3.9	96.97
	250	0.92 ± 0.05	237.00 ± 2.24	0.863	28.31 ± 4.6	96.64

It can be seen that the Nyquist plots depict single capacitive loops appearing as depressed semi-circles with their centers below the real *x*-axis. The observed deviation from the ideal capacitor can be ascribed to the surface heterogeneity occurring due to corrosion. To accurately model the metal–solution interface in such situations, it is a common practice to use a constant phase element (CPE) in the place of a pure double layer capacitor and its impedance can be given by:^44,54^

11 *Z*_CPE_ = *Y*_o_^−1^(j*ω*)^−*n*^

where, *Y*_o_ is a proportionality coefficient, *ω* denotes the angular frequency (*ω* = 2π*f*: having units in rad s^−1^), *n* denotes the phase shift. The computed electrochemical data are listed in Table 2. For the values of *n* = −1, 0 and 1, the CPE is equivalent to the conventional electrical elements, *i.e.*, the inductance (*L*), the resistor (*R*) and the capacitor (*C*) respectively.^3^ Thus, the value of *n* parameter can be taken as a measure of the surface homogeneity. From the data shown in the table, it can be observed that the ‘*n*’ values in the presence of CS–AMT are higher compared to that in its absence. This reveals that the introduction of CS–AMT to the aggressive electrolyte makes the steel surface more homogenous compared to the blank sample which supports the adsorption and protection behavior of the inhibitor.

**Fig. 8:**
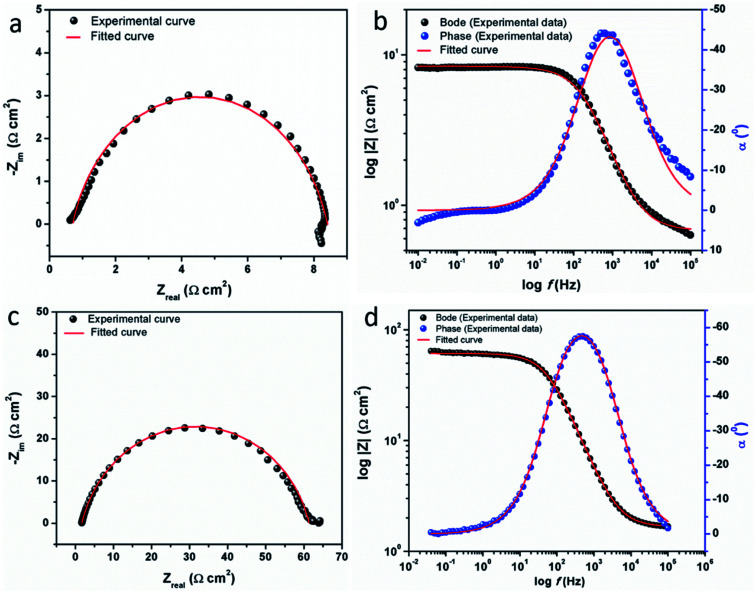
Experimental and fitted EIS data for carbon steel in the absence (a, b) and in the presence (c, d) of CS–AMT.

The fitted EIS curves for the blank steel substrate and in the presence of CS–AMT are shown in Fig. 8a–d. In corrosion inhibition studies, the degree of difficulty in the charge transfer process during corrosion can be measured by the *R*_p_ values wherein a higher *R*_p_ value reflects a decrease in the corrosion rate.^1^ It can be observed that the diameter of the Nyquist semicircles shows an increasing trend with an increase in the concentration of CS–AMT which suggests that the inhibitor molecules get adsorbed on the metal surface leading to the formation of a protective film.^35,55^ The CS–AMT molecules adsorb on the surface of carbon steel and block the sites which are available for corrosive dissolution whereby causing an increment in the *R*_p_ values which is correlated with corrosion inhibitive performance. The polarization resistance (*R*_p_) can be used to calculate the inhibition efficiency as given below:^7,8^12
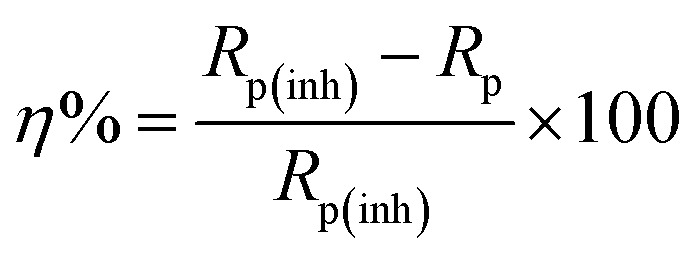
where the terms *R*_p_ and *R*_p(inh)_ represent the polarization resistance without and with the CS–AMT respectively. The values of the double layer capacitance (*C*_dl_) can be estimated as given below:^1,23^

13 *C*_dl_ = *Y*_o_(*ω*_max_)^*n*−1^

where *ω*_max_ represents the frequency at which the imaginary quantity of impedance has gained the maximum (rad s^−1^) value.

The Bode and the phase angle diagrams for the metal samples in the absence and the presence of varying concentrations of CS–AMT are depicted in Fig. 8b. The value of the *n* parameter can be derived from the slope of the Bode plot, and it is related to the extent of deviation from the ideal capacitive behavior. The EIS loop close to the higher frequency region is correlated with the ohmic resistance of the electrolyte between the working electrode and the reference electrode.^49,56^ After this, a linear region between the log|*Z*| *vs.* log *f* values can be observed having a slope approaching 0.9 and the phase angle tending towards −70°. This suggests the characteristic capacitive behavior at intermediate frequencies. An ideal capacitor would show a slope value of −1 and a phase angle value of −90°.^22^ As discussed above, this observed deviation from the ideal capacitive behavior is attributable to the surface roughness and inhomogeneity arising on account of corrosion. However, the gradual approach of slope and the phase angle values to the ideal capacitive behavior may be attributed to slowing down of the rate of dissolution with time which reflects the inhibitive action of CS–AMT.

#### 3.2.3 Potentiodynamic polarization measurements

**Fig. 9:**
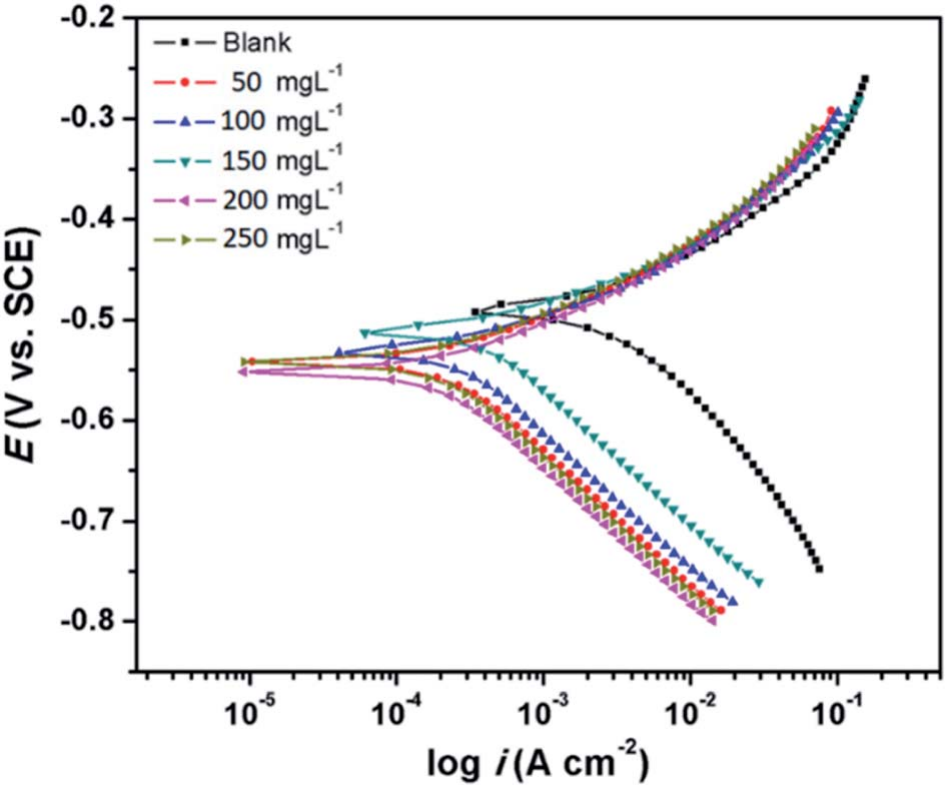
Tafel plots for carbon steel in 1 M HCl in the absence and presence of optimum concentration (200 mg L^−1^) of CS–AMT at 308 K.

The Tafel curves obtained by scanning the potential of the working electrodes without and with the varying concentrations of CS–AMT in the electrolytic solution of 1 M HCl are depicted in Fig. 9. The corresponding electrochemical data, *i.e.* the corrosion current density (*i*_corr_), corrosion potential (*E*_corr_), anodic Tafel slope (*β*_a_) and the cathodic Tafel slopes (*β*_c_) were calculated from the linear regions of the current–potential relationships, and the data are listed in Table 3. The corrosion inhibition efficiency was derived from *i*_corr_ as given below:^1,4,7,23^14
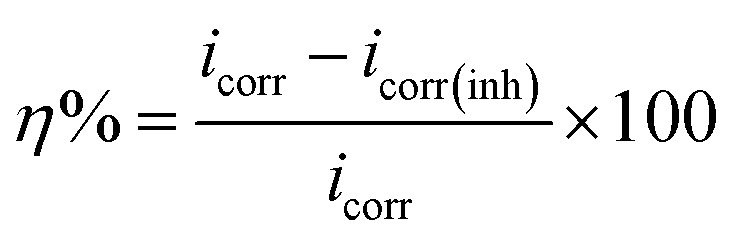
where, *i*_corr_ and *i*_corr(inh)_ represent the corrosion current densities without and with the presence of CS–AMT.

**Table 3:** Polarization data for carbon steel in the absence and presence of different concentrations of CS–AMT

Inhibitor conc. (ppm)	*E*_corr_ (mV *vs.* SCE)	*β*_a_ (mV dec^−1^)	−*β*_c_ (mV dec^−1^)	*i*_corr_ (μA cm^−2^)	*η*%
Blank	−466 ± 2	78.9	129.4	1244	—

**CS–AMT**					
50	−513 ± 2	76.3	111.5	304.5	75.52
100	−521 ± 3	71.1	114.8	195.6	84.27
150	−535 ± 3	69.7	119.3	151.2	87.84
200	−549 ± 4	66.3	118.2	114.3	90.81
250	−538 ± 3	64.4	117.1	123.3	90.09

It can be observed from Fig. 9 and Table 3 that the anodic and cathodic corrosion current densities suppressed in the presence of CS–AMT, suggesting the inhibition of the anodic metal electrodissolution and the cathodic hydrogen evolution. This shows a reduction in the rate of the electrochemical reactions which can be attributed to the adsorption of the inhibitor molecules and the formation of a protective film over the metal surface.^57,58^ It can also be observed that the cathodic currents are suppressed to a greater extent compared to the anodic currents which indicates a cathodic predominance of the CS–AMT.^48^ The cathodic Tafel curves exhibit nearly parallel lines which suggests that there is no change in the mechanism of hydrogen evolution in the presence of inhibitor. The linear portions of the cathodic and the anodic curves were selected and extrapolated to obtain the corrosion potential (*E*_corr_). A slight shift towards the negative direction as observed in the *E*_corr_ values also supports the greater influence of the inhibitor molecules on the cathodic reaction compared to that on the process taking place at the anode. This shift, however, is not significant and the corrosion protection performance can be categorized as a mixed-type inhibition. In addition, the cathodic Tafel branches depict parallel lines (Fig. 9) which suggests that the CS–AMT does not alter the mechanism of hydrogen evolution and indicates that the hydrogen ion reduction takes place mainly *via* a charge transfer mechanism.^59–62^

### 3.3 Surface analysis

**Fig. 10:**
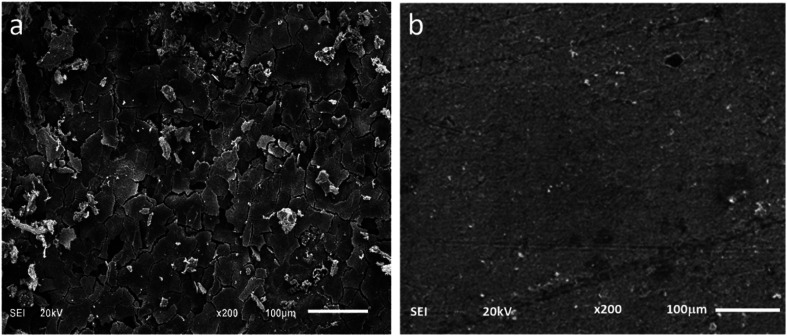
SEM images of carbon steel surface in (a) 1 M HCl, in the absence and (b) in the presence of 200 mg L^−1^ CS–AMT.

The SEM images of the metal surface after immersion in 1 M HCl without and in the presence of the optimum inhibitor concentration (200 mg L^−1^) are displayed in Fig. 10. The carbon steel in absence of inhibitor shows a significant surface damage because of the aggressive acid attack. However, in the presence of CS–AMT, an obvious reduction in the surface damage is shown. This improvement can be ascribed to the adsorption of CS–AMT on the metal surface and the formation of a protective film of CS–AMT thereby isolating the carbon steel surface from the surrounding environment of the corrosive acid.^1,7^

**Fig. 11:**
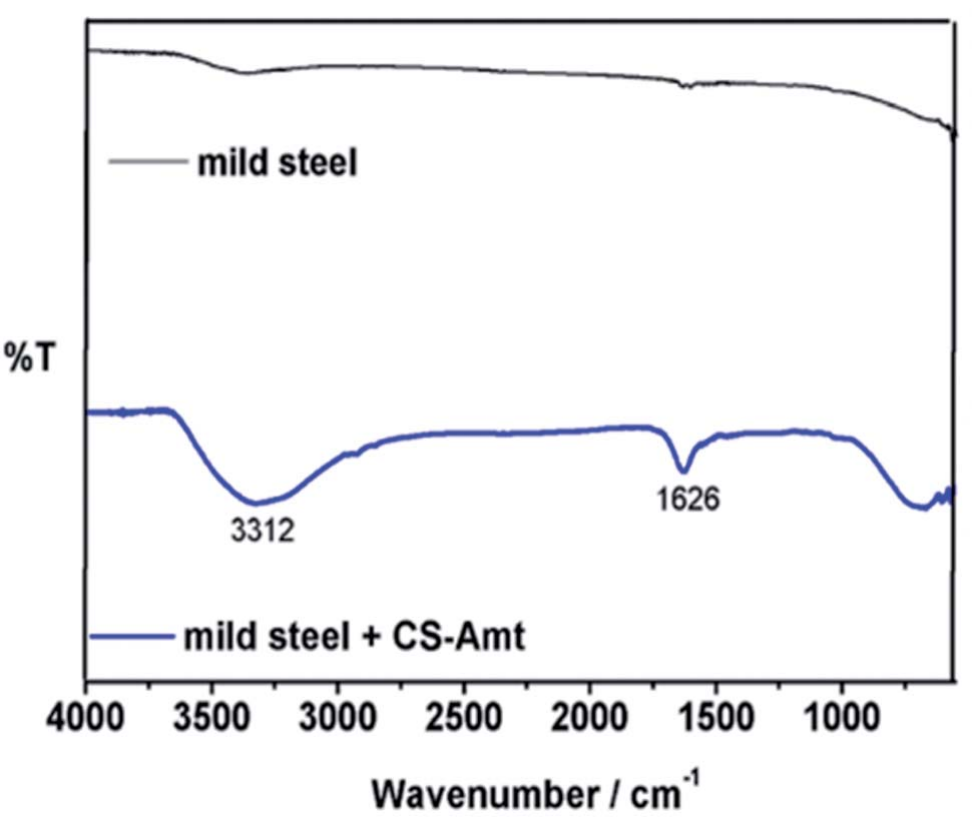
FTIR spectra of carbon steel and carbon steel with CS–AMT after immersion in 1 M HCl.

The FTIR spectra of blank carbon steel substrate and carbon steel substrate in the presence of CS–AMT after 12 h immersion in 1 M HCl solution are depicted in Fig. 11. The spectrum of the synthesized CS–AMT is shown above in Fig. 2, and the related discussion is given in the Experimental section. The FTIR-ATR spectra of inhibited carbon steel shows the distinguishing peaks of pure inhibitor (Fig. 11). However, compared to the band of inhibited carbon steel with that of pure CS–AMT, the shift in the peak suggested that the functional groups help in the effective adsorption of CS–AMT inhibitor on the carbon steel surface.^7,8^

### 3.4 Computational studies

#### 3.4.1 Quantum chemical calculations

The Density Functional Theory (DFT)-based computations can allow an estimation of the reactivity indices of an organic corrosion inhibitor which undergoes adsorption at a metal/electrolyte interface.^34,63,64^ The DFT based quantum chemical calculations are often carried out for obtaining the frontier molecular orbital electron density *ρ*(*r*) in case of corrosion inhibition studies which provides the calculation about the various reactivity parameters.^34^

##### 3.4.1.1 Optimized molecular structures

**Fig. 12:**
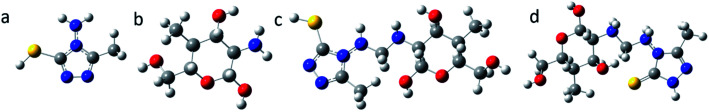
Optimized molecular structures of (a) AMT, (b) CS, (c) CS–AMT and (d) CS–AMT protonated showing the molecular orbital energy gap. The blue color represents Nitrogen, red represents Oxygen, green represents Carbon and white represents Hydrogen.

The optimized molecular structures of AMT, CS and CS–AMT are shown in Fig. 12. In order to understand the influence of functionalization of AMT with CS, a comparative analysis of the reactivity parameters of AMT, CS and a single unit of AMT linked with a single ring of CS is presented. It can be observed that AMT shows a largely planar structure whereas CS shows a non-planar arrangement. This is reflected in the structure of CS–AMT as well where a planar triazole ring is observed. A widely stretched and highly planar arrangement of atoms allows better interaction with the metal surface and hence better adsorption and corrosion inhibition.^23,42^

##### 3.4.1.2 Frontier molecular orbital energies

**Fig. 13:**
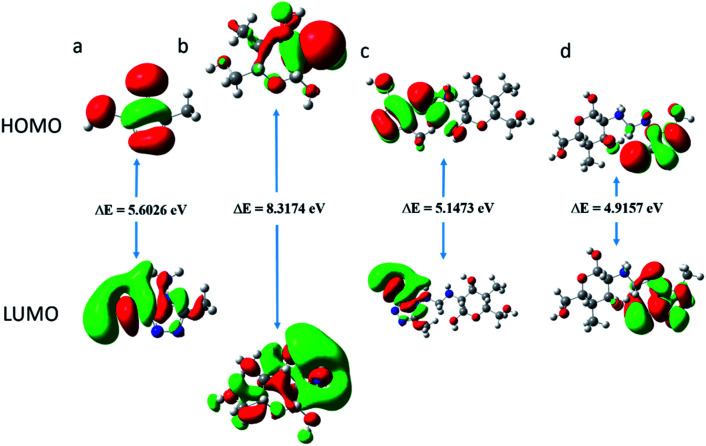
The highest occupied molecular orbital (HOMO); and the lowest unoccupied molecular orbital (LUMO) electron density distribution of (a) AMT, (b) CS, (c) CS–AMT and (d) CS–AMT protonated.

The highest occupied molecular orbital (HOMO) and the lowest unoccupied molecular orbital (LUMO) in a corrosion inhibitor are crucial in estimating its corrosion inhibiting capability. The electron donation tendency can be associated with the HOMO region whereas the region LUMO expresses electron acceptation tendency in a molecule.^44^ In other words, on the basis of the frontier molecular orbital (FMO) theory, the HOMO and the LUMO regions in a corrosion inhibitor represent the active sites which are mainly responsible for the exchange of electrons. The distributions of HOMO/LUMO in AMT, CS and CS–AMT are shown in Fig. 13. The chemical reactivity of CS–AMT can be estimated in terms of the various quantum chemical parameters which can be derived from the energies of the highest occupied molecular orbital (*E*_HOMO_) and the lowest unoccupied molecular orbital (*E*_LUMO_). A high *E*_HOMO_ in an inhibitor indicates a higher electron donation tendency to the metal surface and a lower *E*_LUMO_ value is correlated with a higher capability to accept the electrons. Therefore, a corrosion inhibitor which shows a higher *E*_HOMO_ and lower *E*_LUMO_ can be expected to exhibit better adsorption and therefore, is likely to act as an efficient corrosion inhibitor.^1,23^ The molecular orbital energies can further be related to the ionization potential and the electron affinity according to Koopman's theorem:^34,63^

15 −*E*_HOMO_ = *I*

16 −*E*_LUMO_ = *A*

where, *I* and *A* respectively represent the ionization energy and the electron affinity which can provide the values of the electronegativity and the hardness as shown below:^1,34,63^17
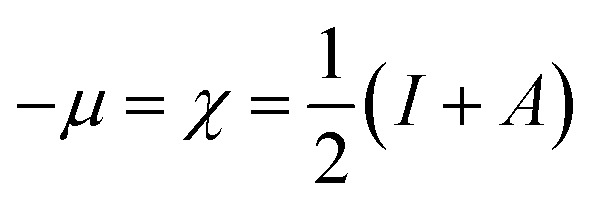
18
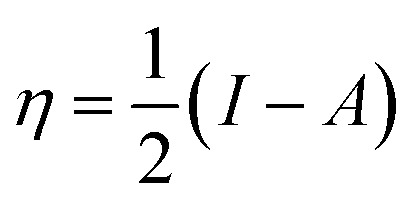
19
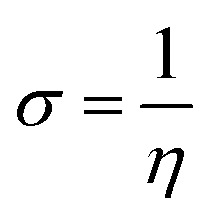
where *μ* defines the chemical potential, *χ* denotes the absolute electronegativity, *η* represents the global hardness and its inverse, *σ* represents the softness. The global hardness is also related to the molecular orbital energy gap, *i.e.* Δ*E*. A stable molecule is likely to exhibit a greater energy gap in comparison to a reactive molecular structure and consequently a greater hardness. This phenomenon is grounded in the rule of maximum hardness: “a molecule arranges itself to be as hard as possible”.^65–67^ Parr *et al.* put forward the global electrophilicity index (*ω*):^68^20
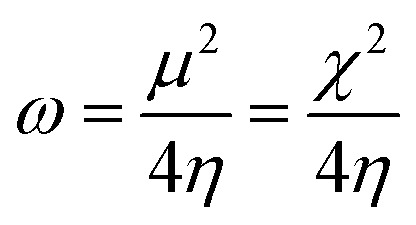


The nucleophilicity (*ε* = 1/*ω*) is the reciprocal of electrophilicity. The *ω* shows the inclination of an inhibitor molecule towards electron acceptance. Therefore, a low value of *ω* (and hence, low *χ*) characterizes a good nucleophile and *vice versa*.

The above-mentioned quantum chemical parameters were calculated and given in Table 4. The data shows that CS has the highest value of *E*_HOMO_ which suggests the highest tendency to donate electrons. CS–AMT shows the lowest value of *E*_LUMO_ suggesting the highest tendency of electron acceptance. The lowest value of Δ*E* in CS–AMT is indicative of its high reactive tendency. The dipole moment (*μ*) was also evaluated which is often used to evaluate the polarity of an inhibitor molecule. It is noteworthy to mention that the net dipole moment can provide the overall polarity of a molecule instead of the polarity of a single bond. The CS–AMT presents the highest value of dipole moment while CS alone shows the lowest dipole moment. However, in literature, there are considerably contrary opinions on directly correlating the dipole moment with the corrosion inhibition efficiency.^69^Table 4 shows that CS–AMT possesses the lowest value of global hardness and hence the highest value of global softness which suggests its high tendency to undergo interaction with the metal surface. Further, it can be observed that the CS–AMT shows the highest value of electrophilicity and the lowest value of nucleophilicity which suggests its higher tendency to accept electrons from the metal surface.

**Table 4:** Calculated quantum chemical parameters

Inhibitors	*E*_HOMO_ (eV)	*E*_LUMO_ (eV)	Δ*E* (eV)	*μ* (Debye)	*I* = −*E*_HOMO_	*A* = −*E*_LUMO_	*χ*	*η*	*σ*	*ω*	*ε*
AMT	−6.0673	−0.4648	5.6026	6.7474	6.0673	0.4648	3.2660	2.8013	0.3569	0.9519	1.0504
CS	−6.5933	1.7241	8.3174	2.2937	6.5933	−1.7241	2.4346	4.1587	0.2405	0.3563	2.8065
CS–AMT	−5.5824	−0.4351	5.1473	9.2652	5.5824	0.4351	3.0088	2.5737	0.3886	0.8794	1.1372
CS–AMT protonated	−6.8148	−1.8991	4.9157	18.4819	6.8148	1.8991	4.3569	2.4579	0.4069	1.9308	0.5179

A corrosion inhibitor, when introduced to an aqueous acid solution, is likely to exhibit a tendency to get protonated and act by adsorbing on the metal surface. Therefore, in addition to the neutral form, the calculation of the reactivity parameters of the protonated form of a corrosion inhibitor also becomes important. The quantum chemical parameters of the protonated CS–AMT are given in Table 4. It is obvious that the protonation of the molecule brings a significant decrease in the *E*_HOMO_ and the *E*_LUMO_ values. The energy gap Δ*E* shows a decrease compared to the neutral form and the global softness shows an increase which indicates that there is an increase in the reactivity of the inhibitor molecules in the protonated form. The electronegativity as well as the electrophilicity index also show higher values suggesting an increased inclination towards acceptance of electrons. The dipole moment also shows an increase in the protonated form.

#### 3.4.2 Fukui indices

The inhibitor molecules undergo adsorption over the metal surface *via* donor–acceptor type (D–A) interaction. This interaction takes place *via* the active sites present in the inhibitor molecules.^70^ In order to locate and analyze the nucleophilic and electrophilic sites present in a studied inhibitor molecule, the analysis of Fukui indices becomes important.^36,70^ The local nucleophilicity and electrophilicity of different sites present on an inhibitor molecule can be determined by the maximum threshold values of *f*_k_^+^ and *f*_k_^−^. Upon electron acceptance, the *f*_k_^+^ measures the changes in the electron density, whereas the *f*_k_^−^ parameter takes into account the changes in electron density upon electron donation by the molecule. High value of *f*_k_^+^ and *f*_k_^−^ are indicative of the greater electron acceptance and electron donation abilities of a molecule respectively.^37,38^ It can be observed from the calculated Fukui indices (vide Table 5) that the sites that are most susceptible for electron acceptance/donation are the N, O and S heteroatoms, followed by some C atoms. For AMT, the favorable sites for electron acceptance, *i.e.*, nucleophilic attack are N1, N2, C3, N4, C5 and S7 having high *f*_k_^+^ values. The sites susceptible for the electrophilic attack, *i.e.*, electron donation are N1, C3, N4, C5 and S7 having high *f*_k_^−^ values. In CS, the favorable sites for electron acceptance are C6, N10 and O11 whereas sites for electron donation are O1, N10 and O11. In CS–AMT, the preferred sites for nucleophilic attack are N14, C16, N17 and C18 while the sites susceptible towards electrophilic attack are O1, N10 and O11.

**Table 5:** Calculated Fukui functions for the studied inhibitor molecules in the neutral form

AMT			CS			CS–AMT		
Atom	*f*_k_^+^	*f*_k_^−^	Atom	*f*_k_^+^	*f*_k_^−^	Atom	*f*_k_^+^	*f*_k_^−^
N1	0.125	0.119	O1	0.008	0.067	O1	0.001	0.092
N2	0.036	0.011	O2	−0.015	0.022	O2	0.001	0.038
C3	0.080	0.085	C3	0.011	−0.001	C3	0.000	0.006
N4	0.064	0.113	C4	0.001	−0.016	C4	−0.001	−0.017
C5	0.122	0.056	C5	−0.013	−0.010	C5	−0.001	−0.010
C6	−0.005	−0.003	C6	−0.040	−0.012	C6	−0.001	−0.011
S7	0.250	0.366	C7	−0.030	−0.038	C7	−0.003	−0.025
N8	0.027	0.029	C8	0.002	−0.001	C8	0.000	−0.002
			O9	−0.009	0.017	O9	0.000	0.020
			N10	−0.068	0.286	N10	0.000	0.150
			O11	−0.076	0.090	O11	0.002	0.069
			C12	−0.010	0.000	C12	0.000	−0.001
						C13	−0.010	−0.014
						N14	0.147	0.026
						N15	0.031	−0.004
						C16	0.071	0.013
						N17	0.066	0.025
						C18	0.129	0.011
						C19	−0.004	−0.002
						S20	0.202	0.031
						N21	0.021	0.020

From the analysis of Fukui indices, it can be observed that the sites most susceptible towards electron donation are the heteroatoms N and S. This suggests that the functional groups *viz.* –NH–, C=S and hence, the heterocyclic triazole ring are the major centers involved in the adsorption of CS–AMT over the metal surface. Moreover, the calculated Fukui indices show accordance with the frontier molecular orbital electron density distributions in CS–AMT as discussed above.

#### 3.4.3 Molecular dynamics simulations

**Fig. 14:**
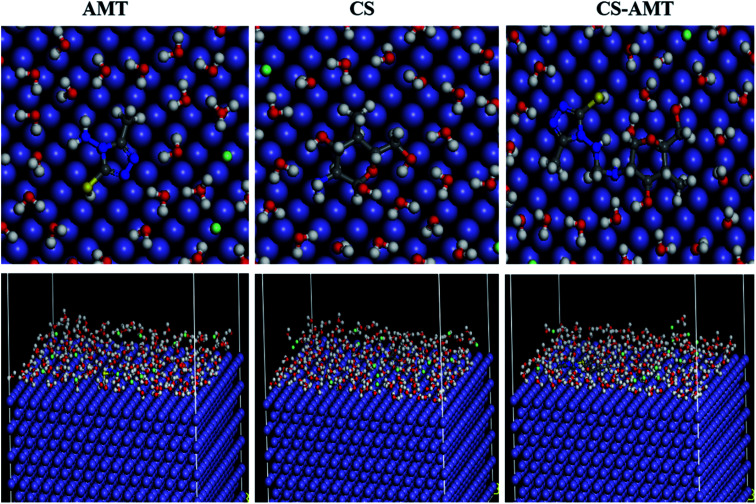
Top and side views of the final adsorption of AMT, CS and CS–AMT on the Fe (110) surface in a solution.

Molecular dynamics (MD) simulation is a modern tool for investigating the inhibitor adsorption on a metal surface.^1^ In the present study, to understand the interaction of AMT, CS and CS–AMT with the Fe (1 1 0), MD simulation studies were undertaken with consideration of all the concerned species (such as H_2_O, H_3_O^+^, Cl^−^ and Fe surface) which are involved in the corrosion. An appropriate and authentic configuration of surface adsorbed inhibitor molecules was obtained using MD simulation. The side and top views of the equilibrium adsorption configurations of surface adsorbed inhibitors are presented as Fig. 14 which elucidated that inhibitor molecules tend to acquire more or less planar orientation to favor their adsorption on the Fe (1 1 0) surface. This particular orientation provided maximum contact of inhibitor molecules with metal surface leaving the minimum surface area to be attacked by aggressive corrosive species. The binding strength of AMT, CS and CS–AMT on the surface of Fe is evaluated by MD simulations which establishes a correlation between the theoretically determined binding energies of the abovementioned inhibitors molecules with the experimentally determined inhibition efficiencies. Firstly, the geometry optimization of the studied molecules was performed as discussed earlier.^36–38^ During the optimization of geometry, the atomic coordinates were adjusted using the COMPASS force field until the total energy arrives at a minimum value, after which the inhibitor was put on the Fe (1 1 0) surface to figure out the most appropriate configuration of adsorption. The system arrives at equilibrium when the temperature and energy reach a balance, and afterward the interaction energy (*E*_interaction_) between the surface of Fe (1 1 0) and the inhibitors was calculated.

The computed interaction energies of the adsorption systems are in the order −84.876, −114.513 and −191.332 kcal mol^−1^ for AMT, CS and CS–AMT, respectively. The high negative interaction energy values are attributable to strong adsorption of the molecules on the Fe surface.^37,39^ Valuable information regarding the adsorption behavior of inhibitors can be extracted from the interaction energy (*E*_interaction_) or binding energy (*E*_binding_) values as shown in Table 6. The results of *E*_interaction_ indicated that the combination of CS and AMT interacts more strongly than AMT and CS alone with the Fe (1 1 0) surface. Thus, it can be concluded that the theoretical results agreed well with the experimental results. Further, the ability of adsorption can be determined from the binding energy of the molecules with the Fe surface. The higher the binding energy, the stronger will be the adsorption on the metal surface.^1,36,38,71^ From Table 6, it can be observed that the binding energy with the Fe surface follows the order: CS–AMT > CS > AMT. Thus, CS–AMT shows a better adsorption ability on the Fe surface than CS and AMT.

**Table 6:** Output obtained from MD simulation for adsorption of inhibitors on Fe (110) surface

System	*E*_interaction_ (kcal mol^−1^)	*E*_binding_ (kcal mol^−1^)
Fe + AMT	−84.876	84.876
Fe + CS	−114.513	114.513
Fe + CS–AMT	−191.332	191.332

## 4. Conclusions

A convenient synthesis of triazole functionalized chitosan is reported for the first time. The synthesized chitosan derivative was thoroughly characterized using FTIR and NMR. The compound was used as a novel and environment-friendly inhibitor against corrosion of carbon steel in 1 M hydrochloric acid. The results of gravimetric measurements showed that the new inhibitor showed 92.6% efficiency at a concentration of 200 mg L^−1^. The CS–AMT adsorption on the carbon steel obeyed the Langmuir isotherm while exhibiting physical as well as chemical adsorption. The EIS data revealed successive increments in the polarization resistance with increasing concentrations of the inhibitor. The results of Tafel analysis revealed that the inhibitor shows a mixed type behavior having cathodic predominance. The SEM confirmed the improvement in surface homogeneity of carbon steel in the presence of CS–AMT. The results of the DFT analysis supported the observations of the experimental methods. Fukui indices showed that the inhibitor adsorbs over the carbon steel surface with heteroatoms. Molecular dynamics simulation showed that the inhibitor showed increased binding energy of the Fe-inhibitor system suggesting ease of adsorption of inhibitor over the metal surface.

## Conflicts of interest

The authors declare no conflict of interest.

## Supplementary Material

RA-009-C9RA00986H-s001
