# Supplementary material for: Triazole-modified chitosan: a biomacromolecule as a new environmentally benign corrosion inhibitor for carbon steel in a hydrochloric acid solution
Source: RSC Adv. 2019 May 14;9(26):14990–5003. doi: 10.1039/c9ra00986h (PMC9064206; doi:10.1039/c9ra00986h)
Supplement: RA-009-C9RA00986H-s001 [file RA-009-C9RA00986H-s001.pdf]

Electronic Supplementary Material (ESI) for RSC Advances.
This journal is © The Royal Society of Chemistry 2019

Triazole-modified Chitosan: A biomacromolecule as a new environmentally benign corrosion inhibitor for carbon steel in a hydrochloric acid solution

Dheeraj Singh Chauhan[1]*, M.A. Quraishi[1]**, A.A. Sorour[1], S.K. Saha[2], P. Banerjee[2]

[1]*Center of Research Excellence in Corrosion, Research Institute, King Fahd University of Petroleum and Minerals, Dhahran 31261, Saudi Arabia*

[2]*Surface Engineering & Tribology Group, CSIR-Central Mechanical Engineering Research Institute, Mahatma Gandhi Avenue, Durgapur 713209, West Bengal, India.*

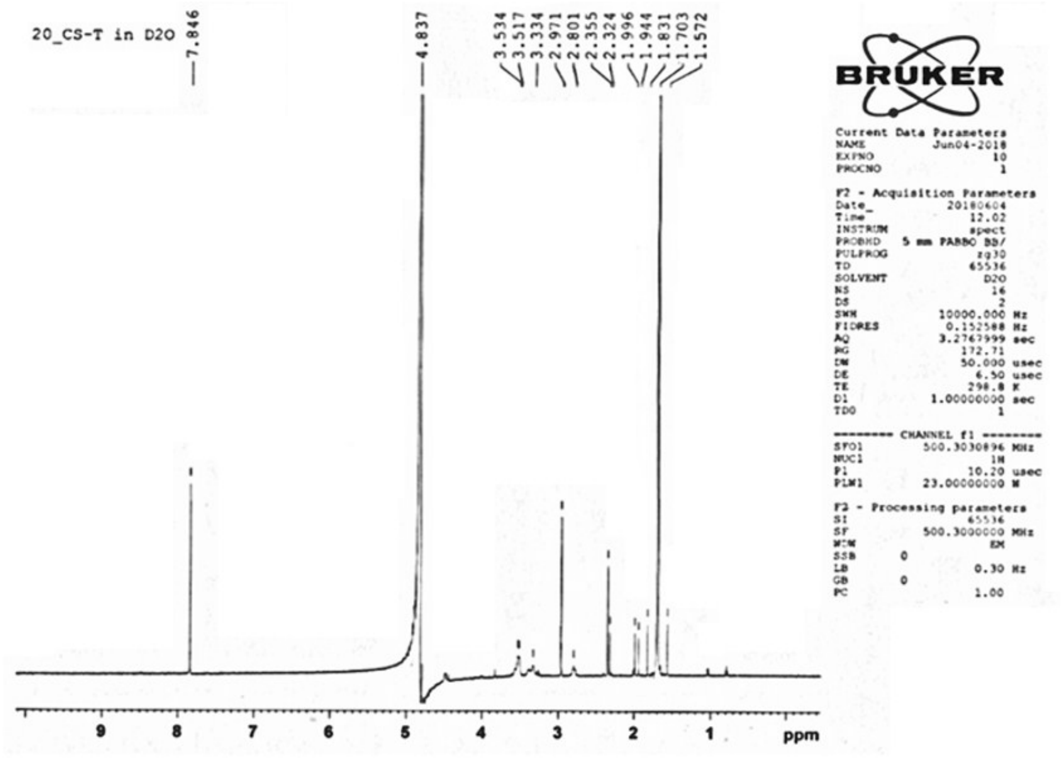

Figure S1 ^{1}H NMR spectrum of CS-AMT
